# The incidence of clinically diagnosed chronic obstructive pulmonary disease in patients with spondyloarthritis compared to matched population-based references: a register-based study

**DOI:** 10.1007/s10067-026-08382-7

**Published:** 2026-09-02

**Authors:** Sofia Exarchou, Ankita Sharma, Petros Zamout, Carl Turesson

**Affiliations:** Department of Clinical Sciences Malmö, Rheumatology, Lund University, Jan Waldenströms Gata 1B, 205 02 Malmö, Sweden

**Keywords:** COPD, Epidemiology, Incidence, Spondyloarthritis, Sweden

## Abstract

**Introduction/Objective:**

To estimate and compare the incidence of chronic obstructive pulmonary disease (COPD) in patients with spondyloarthritis (SpA) to matched population-based references.

**Methods:**

Patients with ≥ 1 SpA-diagnosis from an outpatient department of rheumatology/internal medicine (≥ 17 years at diagnosis) between January 1, 2000, and June 30, 2005, were identified in a regional healthcare register. From the same register, five controls without SpA-diagnosis were matched to each SpA-case on age at the first ever SpA-diagnosis for the case, municipality of residence at this time, and sex. SpA-cases and controls were followed from Jan 1, 2006, until first occurrence of either COPD (diagnosed in primary or secondary inpatient/outpatient care), rheumatoid arthritis diagnosis, moving from the region, death, or Dec 31, 2018. The incidence rates of COPD in SpA-cases and controls, overall and by age-groups and sex, and the corresponding incidence rate ratios, were estimated.

**Results:**

A total of 1083/4950 SpA-cases and controls were followed in the present study for 11,790.9/58,876.4 person-years, with 38/220 incident COPD-cases respectively. The estimated incidence of COPD in SpA-cases was 3.22 (95% CI 2.28–4.42) per 1000 person-years, with an incidence rate ratio compared to controls of 0.86 (95% CI 0.59–1.22). Similarly, there were no significant differences in the incidence of COPD in patients with SpA compared to controls in any age group and in both sexes.

**Conclusions:**

In this register-based cohort study the incidence of COPD in patients with SpA was similar to that in matched population-based references, overall and after stratification for sex and age-groups.

**Table:** 

**Key Points** *• Spondyloarthritis (SpA) has been associated with comorbidities, e.g. cardiovascular disease. However, evidence regarding the relation between SpA and chronic obstructive pulmonary disease (COPD) is limited.* *• To our knowledge, this is the first study to investigate the incidence of COPD in a population-based cohort of patients with SpA in general and compare it to the background population.* *• Using a regional register of primary and specialized care, we found no difference in COPD incidence between those diagnosed with SpA and matched population-based references, overall and after stratification for sex and age group.* *• The results of the present study suggest that there may not be an association between SpA and COPD. However, further studies in other countries and settings are needed.*

**Supplementary Information:**

The online version contains supplementary material available at https://doi.org/10.1007/s10067-026-08382-7.

## Background

Spondyloarthritis (SpA) is a heterogeneous group of chronic inflammatory diseases that share genetic, pathophysiological and clinical features [1]. SpA is classified as either axial SpA (axSpA) or peripheral SpA (pSpA) depending on the predominant musculoskeletal manifestations (axial or peripheral) [2, 3]. AxSpA patients are further classified as radiographic axSpA (r-axSpA), historically known as ankylosing spondylitis (AS), or non-radiographic axSpA (nr-axSpA), based on the presence or absence of radiographic evidence of sacroiliitis [4–6]. SpA frequently occurs together with other inflammatory extra-musculoskeletal manifestations, such as psoriasis, anterior uveitis, and inflammatory bowel disease [1].

Patients with SpA are also at higher risk of other medical conditions compared to the general population [7, 8]. The presence of comorbidities contributes to an increased disease burden, more complicated therapeutic decisions, worse treatment outcomes and the increased mortality risk of the disease [9–12]. Cardiovascular disease, osteoporosis, infections and depression are all well-known to be more prevalent in patients with SpA than in the general population [7, 8]. Restrictive pulmonary function impairment, mainly extrinsic, due to thoracic involvement is well described in SpA [13]. By contrast, there is limited information on the relation between SpA and chronic obstructive pulmonary disease (COPD).

Register-based studies from Israel [14] and Taiwan [15] have suggested an increased prevalence of COPD in patients with AS compared to the general population. In our register-based study from the southern region of Sweden, including data from both primary and specialised care, the prevalence of COPD in patients with SpA did not differ significantly from the COPD prevalence in the general population overall, in men or women, in different age groups and after using different definitions of COPD [16]. An increased incidence of COPD has been reported in a study of patients hospitalized with AS in Sweden [17]. In a study from UK using self-reported diagnoses, participants with AS were also at increased risk of developing COPD [18]. However, to our knowledge there have been no studies estimating the incidence of COPD in patients with SpA in general.

COPD is a complex and heterogeneous pulmonary condition resulting from airways inflammation and remodelling, leading to poorly reversible airflow obstruction, and often associated with parenchymal destruction and the development of emphysema [19]. COPD is the result of interplay between risk exposures, such as cigarette smoke and air pollutants, and genetic, developmental, and social factors [19]. COPD ranks among the three leading causes of morbidity and mortality globally, causing a huge burden that is increasing despite efforts to improve modifiable risk factors [20, 21]. An increased risk of COPD has been found in patients with rheumatoid arthritis (RA) [22], probably partly due to smoking as a common risk factor, but also likely related to effects of chronic inflammation [23].

Knowledge of the incidence of COPD in patients with SpA will help us to understand the inflammatory nature of SpA and may suggest shared inflammatory and immunologic pathways between the diseases. Information on which comorbidities are overrepresented among patients with SpA would also encourage improved patient management and subsequently reduced morbidity and mortality in SpA, as well as increased awareness among healthcare providers and resource allocation within the public health system.

The aim of the present study was therefore to estimate the incidence of COPD in a population-based cohort of patients with SpA and compare it to the general population.

## Methods

### Study setting and data sources

This observational study was based on a comprehensive linkage of data from different population-based databases with the use of the personal identity number, a ten-digit number that is unique to every permanent Swedish resident.

The Skåne Healthcare Register (SHR) was the main data source in the present study. The SHR is an administrative healthcare register that contains information from electronic medical records and administrative databases on each single healthcare contact with inpatient, primary and secondary outpatient care in the Skåne region since 1998. During the 5-year period 2012–2016, 97% of the general population in the region had at least one registered healthcare contact [24]. For inpatient care, the proportion of consultations that have an assigned International Classification of Diseases (ICD)−10 diagnosis has been close to 100% over the whole period [24]. For secondary outpatient care, the proportion of consultations with a registered diagnosis has been high and increasing since 2000 [24]. The proportion of consultations with assigned diagnosis has been lowest within primary care, reaching ≥ 80% for physician visits after 2004, and close to 100% in 2017 [24]. For the present study, ICD-10 diagnostic codes from inpatient care, primary care and secondary outpatient care were retrieved from the SHR to identify patients with a diagnosis of SpA, as well as incident diagnoses of COPD (for ICD codes used, see Online Resource 1). Furthermore, individuals with SpA-related extra-musculoskeletal manifestations (psoriasis, anterior uveitis, inflammatory bowel disease) and other selected general comorbidities (cardiovascular disease, arrythmia, venous thromboembolism and diabetes mellitus) before the start of follow-up among patients with SpA and general population comparators were identified using ICD-10 codes (for ICD codes used, see Online Resource 2).

To identify drug treatment with Non-steroid anti-inflammatory drugs (NSAIDs), oral glucocorticoids, and Disease Modifying Anti-Rheumatic Drugs (DMARDs) during the year prior to follow-up start, we used data from the National Prescribed Drug Register (PDR), which holds information on all prescribed drugs dispensed by Swedish pharmacies since July 1 st, 2005, with national coverage close to 100% (for Anatomical Therapeutic Chemical (ATC) codes used, see Online Resource 3) [25]. Since intravenous biologic DMARDs (bDMARDs) administered in hospital are not normally registered in PDR, the Swedish Rheumatology Quality Register (SRQ) was also used as an auxiliary source for such treatments. SRQ is a national quality control register containing clinical information on diagnosis, antirheumatic treatments, and follow-up for patients with rheumatic diseases in Sweden since 1999 and captures approximately 86% of administered bDMARDs for SpA [26].

Information on country of birth, places of residence, and date of death for SpA patients and controls was retrieved from the Total Population Register (TPR), which is maintained by Statistics Sweden (SCB) [27].

### Study population and follow-up

**Fig. 1:**
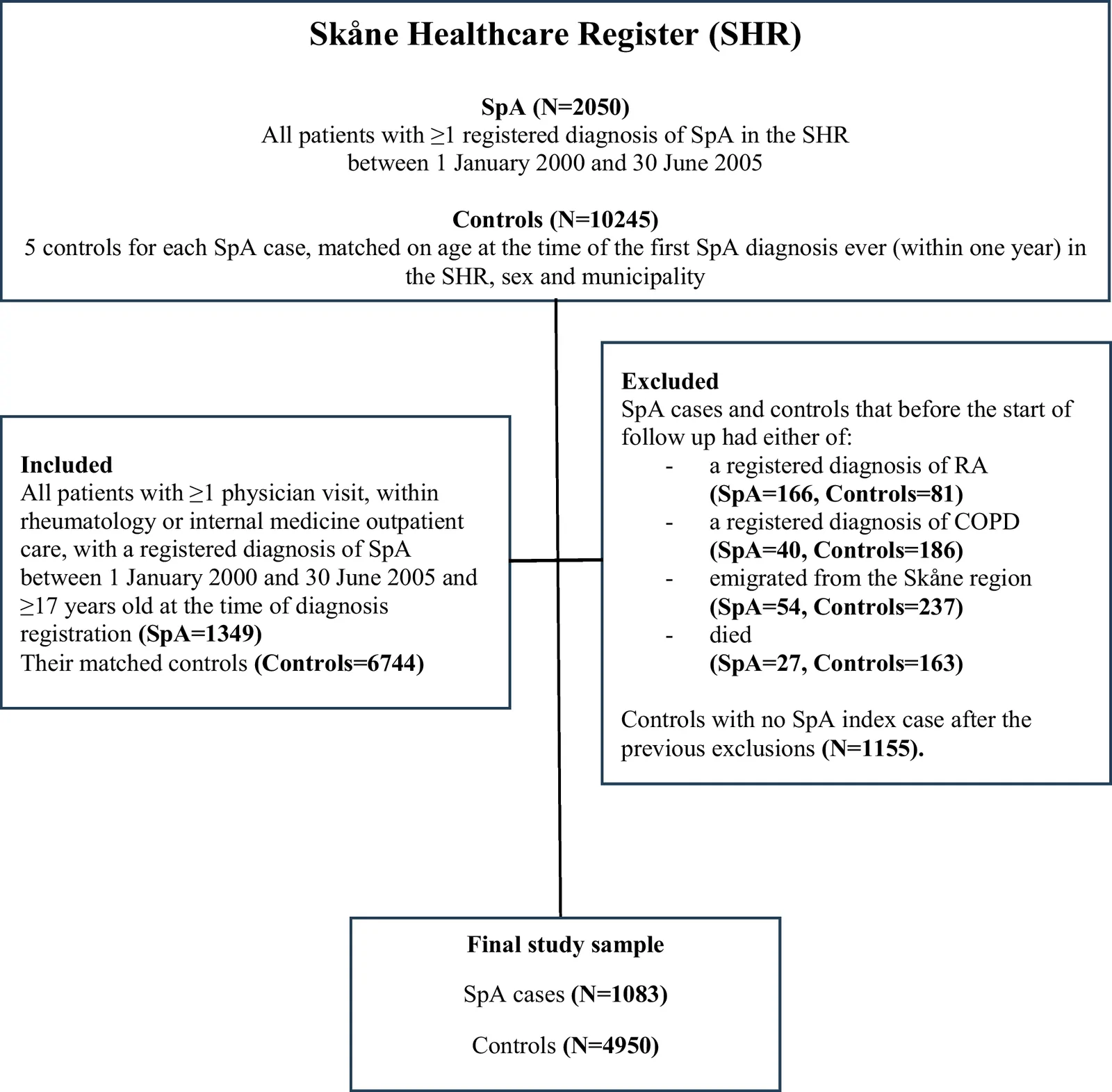
The identification process of study population

The identification of study population is described schematically in Fig. 1. A cohort of patients with SpA was identified from the SHR. Eligible patients had at least one physician visit, within rheumatology or internal medicine outpatient care, reporting an ICD-10 code corresponding to a diagnosis of SpA (M459, M460, M461, M468, M469) between January 1, 2000, and June 30, 2005. The diagnostic codes were chosen to best reflect the entities axSpA (radiographic and non-radiographic) and undifferentiated peripheral SpA. Only those 17 years old or older at the time of diagnosis registration in the SHR were included in the study.

A cohort of matched population-based reference subjects (controls) was identified from the SHR, among those with at least one contact with the healthcare system at any time during the study period and no diagnosis of SpA. The rationale for using the SHR as the source for the reference subjects was to recruit a control group with a propensity to seek health care similar to the cases, as previously discussed [28]. For each SpA case, five controls were matched on age at the time of first SpA diagnosis ever for the case (within 1 year), municipality of residence at this time, and sex.

To strengthen the case definition, to reduce diagnostic misclassification and to avoid overlapping inflammatory arthritis diagnoses, SpA cases and controls with a physician visit reporting an ICD-10 code for rheumatoid arthritis (RA) (M05, M06 or M08) as a diagnosis before the start of follow up were excluded (SpA = 166, Controls = 81). Further exclusion criteria for both SpA and control cohorts included a COPD diagnosis (SpA = 40, Controls = 186), emigration from the Skåne region (SpA = 54, Controls = 237) or death (SpA = 27, Controls = 163) before start of follow-up. Controls with no SpA case after the previous exclusions were also excluded (*N* = 1155).

The follow-up period of both SpA cases and controls started at the 1 January 2006 and continued until first occurrence of the outcome (COPD), diagnosis of RA, moving out from Skåne region, death or 31 December 2018, whichever occurred first.

COPD, the outcome of interest, was defined as the first occurrence of corresponding ICD-10 codes (see Online Resource 1) as a diagnosis in the SHR.

In a sensitivity analysis, we required ≥ 1 visit with a diagnosis of COPD at a specialized lung medicine unit for the definition of the outcome. In addition, we performed a further sensitivity analysis where dispensation of ≥ 1 prescribed drug treatment (for ATC codes used in the PDR, see Online Resource 4), in addition to ≥ 1 with a diagnosis of COPD at any provider, was included in the definition of COPD.

### Statistical analysis

Demographics, comorbidities and medications are presented for SpA cases and controls at the start of follow-up period. Prevalence estimates for general comorbidities at the index date in patients with SpA and controls were calculated, with 95% confidence intervals (CI) estimated using the Poisson distribution ratio.

Incidence rates (IRs) of COPD per 1000 person-years at risk in patients with SpA and controls were calculated by dividing the number of incident COPD events with the person years at risk in each group, overall, by sex and by age-group, i.e., younger (17–50 years), middle aged (50–70 years) and older (> 70 years) individuals. Incidence rate ratios (IRRs) for COPD in patients with SpA compared with controls were also calculated, overall, by sex and age-group. 95% CI for IRs and IRRs were determined using the Poisson distribution ratio.

IRs and IRRs were estimated in the same manner in sensitivity analyses using alternative definitions of COPD.

Statistical analyses were performed by SPSS (version 30).

### Ethics

The study was approved by the Swedish Ethical Review Authority (September 23, 2000; registration number 2020–04041, with amendment on February 16, 2021; registration number 2021–00717). The Ethical Review Authority decided that individual informed consent was not necessary for this study.

## Results

### Patients with SpA and controls

A total of 1083 SpA cases and 4950 controls were included in the present study (Fig. 1, Table 1). Most of SpA patients and controls were male (58%) with a mean age of 48 years and with mainly Swedish origin (Table 1). Patients with SpA had higher prevalence of comorbid conditions at the start of follow-up, i.e., cardiovascular disease, arrhythmia and thromboembolic disease, whereas they were less likely to have diabetes mellitus (Table 1). However, confidence intervals for the prevalence estimates were overlapping (Online Resource 5).

**Table 1:** Patients with spondyloarthritis and controls. Demographics and comorbidities

	SpA	Controls
*N*	1083	4950
Male sex; *n* (%)	628 (57.9)	2861 (57.7)
Age (years); mean (SD)	48.0 (13.0)	48.0 (12.7)
***Country of birth; n (%)***		
Sweden	954 (88.0)	4480 (90.5)
Scandinavia except Sweden	25 (2.3)	74 (1.4)
Europe except Scandinavia	77 (7.1)	236 (4.7)
Other	27 (2.4)	160 (3.2)
***Comorbidities; n (%)***		
Cardiovascular disease	73 (6.7)	311 (6.2)
Diabetes mellitus	28 (2.5)	196 (3.9)
Venous thromboembolism	23 (2.1)	95 (1.9)
Arrhythmia	35 (3.2)	143 (2.8)
***Treatment (ever); n (%)***		
Systemic glucocorticosteroids	133 (12.2)	58 (1.1)
Non-steroidal anti-inflammatory drugs	951 (87.8)	3359 (67.9)

### Characteristics of patients with SpA, by the presence of incident COPD

Among the SpA cases, 38 had an incident COPD diagnosis during the follow-up period. Compared to those never registered with a COPD diagnosis, these were older and more likely to be male and have other than Swedish origin (Table 2). Considering the related extra-musculoskeletal manifestations, those with incident COPD were more likely to have suffered from anterior uveitis, but they were less likely to have psoriasis and inflammatory bowel disease. Further, they also had numerically more comorbid diseases (with overlapping confidence intervals, see Online Resource 6) and were to a lesser extent treated with DMARDs (Table 2).

**Table 2:** Patients with spondyloarthritis by incidence of chronic obstructive pulmonary disease. Demographics, comorbidities, SpA manifestations and treatment

	No COPD	COPD
*N*	1037	38
Male sex; *n* (%)	597 (57.5)	28 (73.6)
Age (years); mean (SD)	47.6 (13.0)	57.7 (10.8)
***Country of birth; n (%)***		
Sweden	920 (88.7)	29 (76.3)
Scandinavia except Sweden	23 (2.2)	0
Europe except Scandinavia	69 (6.7)	7 (18.4)
Other	25 (2.4)	2 (5.2)
***SpA related extra-musculoskeletal manifestations; n (%)***		
Anterior uvetis	234 (22.5)	10 (26.3)
Psoriasis	68 (6.5)	0
Inflammatory bowel disease	63 (6.0)	2 (5.2)
***General comorbidities; n (%)***		
Cardiovascular disease	67 (6.4)	5 (13.1)
Diabetes mellitus	26 (2.5)	1 (2.6)
Venous thromboembolism	20 (1.9)	1 (2.6)
Arrhythmia	34 (3.2)	1 (2.6)
***Treatment (ever); n (%)***		
Methotrexate	89 (8.5)	1 (2.6)
Sulfasalazine	84 (8.1)	4 (10.5)
Other csDMARD	8 (0.7)	0
TNF inhibitor	77 (7.4)	1 (2.6)
JAK inhibitor	0	0
IL-17 inhibitor	0	0
PDE4i	1 (0.1)	0
Any csDMARD	168 (16.2)	5 (13.1)
Any b/ts DMARD	78 (7.5)	1 (2.6)
Any DMARD treatment	205 (19.7)	6 (15.7)
Glucocorticosteroids	125 (12.0)	5 (13.1)
Non-steroidal anti-inflammatory drugs	909 (87.6)	35 (92.1)

*COPD* chronic obstructive pulmonary disease, *DMARDs* Disease Modifying Anti-Rheumatic Drugs, *csDMARDs* conventional synthetic Disease Modifying Anti-Rheumatic Drugs, *tsDMARDs* targeted synthetic Disease Modifying Anti-Rheumatic Drugs, *bDMARDs* biological Disease Modifying Anti-Rheumatic Drugs

### Incidence of COPD in patients with SpA and controls

A total of 258 incident COPD cases were identified in the study population, 38 among the SpA cases and 220 among the controls, during 11,790.9 and 58,876.4 person-years of follow-up respectively. The estimated incidence of COPD in patients with SpA was 3.22 (95% CI 2.28–4.42) per 1000 person-years at risk (pyr). Incidence rates of COPD were similar for patients with SpA and controls overall, with an estimated IRR of 0.86 (95% CI 0.59–1.22). Similarly, there were no significant differences in the incidence of COPD in patients with SpA compared to controls in any age group (Table 3). In both SpA cases and controls, the incidence of COPD increased with age, with highest incidence in those 70 years of age or older. Compared to controls, the incidence of COPD for patients with SpA was numerically lower in the two younger groups, but higher in the group aged ≥ 70 years, although differences were not statistically significant (Table 3). The incidence of COPD was higher in men than in women in both patients with SpA and controls (Table 3). Among SpA cases the incidence of COPD was twice as high in men (IR 4.08; 95% CI 2.71–5.90) compared to women (IR 2.03, 95% CI 0.97–3.73). COPD incidence was slightly, but not significantly higher than in controls for men with SpA [IRR 1.04 (95% CI 0.66–1.56)], whereas the reverse was seen for women (Table 3). After stratifying for sex, the incidence of COPD was still higher in the oldest age-group of both patients with SpA and controls, except for female patients with SpA that did not have any incident COPD case in this age group (Online Resource7).

**Table 3:** Incidence rates and incidence rate ratios for chronic obstructive pulmonary disease in patients with spondyloarthritis and controls

	SpA cases with incident COPD	SpA cases Person-yrs at risk	Controls with incident COPD	Controls Person-yrs at risk	SpA	Controls	COPD incidence ratio (95% CI)
					COPD incidence rate/1000 person-yrs at risk (95% CI)	COPD incidence rate/1000 person-yrs at risk (95% CI)	
Total	38	11,790.9	220	58,876.4	3.22 (2.28–4.42)	3.74 (3.26–4.26)	0.86 (0.59–1.22)
17–49	4	4956.0	21	23,956.7	0.81 (0.22–2.07)	0.88 (0.54–1.34)	0.92 (0.23–2.78)
50–69	22	5556.5	141	28,418.8	3.96 (2.48–5.99)	4.96 (4.18–5.84)	0.80 (0.48–1.25)
70 +	12	1276.9	58	6494.8	9.40 (4.86–16.42)	8.93 (6.78–11.54)	1.05 (0.51–1.97)
Men	28	6861.0	133	33,771.0	4.08 (2.71–5.90)	3.94 (3.30–4.67)	1.04 (0.66–1.56)
Women	10	4929.9	87	25,066.4	2.03 (0.97–3.73)	3.47 (2.78–4.28)	0.58 (0.27–1.12)

IRs were slightly lower in the sensitivity analysis where a diagnosis of COPD by a lung specialist was required (3.14 per 1000 pyr with 95% CI 2.21–4.32 in patients with SpA and 3.69 with 95% CI 3.21–4.21 in controls), with no significant difference between patients with SpA and controls (estimated IRR 0.85; 95% CI 0.58–1.21).

IRs were also slightly lower in the sensitivity analysis where treatment for COPD was a required part of the COPD definition (estimates with 95% CI per 1000 pyr of 2.80 (1.93–3.93) in patients with SpA and 3.29 (2.85–3.79) in controls), but the IRR was similar to the main analysis (0.85; 95% CI 0.57–1.23).

## Discussion

In this register-based cohort study the incidence of COPD, diagnosed in either primary or specialized care, was 3.2 per 1000 pyr in patients with SpA and similar to that in age-, sex- and municipality-matched controls without SpA. The incidence estimates did not differ significantly between patients with SpA and controls in analyses stratified by sex and pre-specified age-groups.

The incidence of COPD increased with age, irrespective of the presence of SpA diagnosis, with higher incidence in those 70 years old or older. This is in harmony with the results of a large prospective population-based cohort study (the Rotterdam Study) which showed that the incidence of COPD increased with the age and peaked at the age of 80 in men and 75 in women [29]. Patterns for the comparison of incidence rates between SpA and controls were largely similar across age groups.

Incident COPD was more frequent in men than in women, both among patients with SpA and controls, with double incidence estimates in men compared to women in the SpA population. This is in accordance with the results of previous studies indicating that that the incidence of COPD is higher in men than in women in the general population [29, 30]. This may reflect higher smoking rates or greater occupational exposure to other risk factors for COPD in men [31, 32]. However, underdiagnosis or misdiagnosis of COPD (e.g., asthma) in women may also contribute to the observed lower incidence in women [33, 34].

The incidence of COPD in SpA is less studied compared to the prevalence. Our recent study found no significant difference in COPD prevalence among SpA patients compared to controls, which contrasts with some older studies [14, 15]. The present study is, to our knowledge, the first addressing the incidence of COPD in patients with SpA in general. An Israeli study (4076 AS cases matched with 4076 controls) found AS to be independently associated with COPD, with an OR of 1.22 after adjusting for age, sex and smoking [14], but the cross-sectional study design precludes the determination of a causal relationship between AS and COPD. Our results indicating similar incidence of COPD in patients with SpA and controls differ from one Swedish Hospital Discharge Register study (1964–1999) [17] that showed an increased incidence for COPD in patients hospitalized with AS compared to the general population, with a standardized incidence ratio of 1.72 (95% CI 1.23–2.33), likely reflecting the higher comorbidity burden in patients that need inpatient care. In contrast to our findings, a more recent study from UK using self-reported diagnoses for both AS and COPD showed that participants with AS were at increased risk of developing COPD with a HR (95% CI) of 2.0 (1.3, 3.1) [18]. However, the retrospective design and self-reported type of exposure and outcome, including date of diagnoses, may have affected the results of that study.

As patients with COPD were older and more often men, it follows that those with COPD and SpA had more comorbidities, in particular from cardiovascular disease, compared to other patients with SpA. The lower proportion receiving TNF inhibitors may reflect less active treatment of older patients. The higher percentage with non-European origin in the SpA-COPD group may be due to differences in smoking or other exposures.

To our knowledge, this is the first study estimating the incidence of COPD in patients not only with AS but with SpA in general. The assessment of COPD using prospectively recorded data from a longitudinal register with high coverage of primary and specialized care visits in the same setting for SpA cases and controls is a strength of this study. As there are no major differences in the diagnosis and registration of both SpA and COPD in Sweden, such factors should not affect generalizability on the national level. However, differences in the prevalence of HLA-B27 and in SpA phenotypes as well as other ethnic factors and environmental exposures might affect estimates in other parts of Sweden and other populations.

A limitation of the present study that we need to acknowledge is the lack of validation of the SpA and COPD diagnoses in the SHR. However, only patients with an SpA diagnosis from a rheumatology or internal medicine department were included in the present study and good validity of SpA diagnoses, overall and for axSpA specifically, at rheumatology departments in the same region has been demonstrated [35, 36]. A good validity of ICD diagnoses for AS and undifferentiated SpA from rheumatology or internal medicine specialist units was also found nationally in Sweden, without geographical differences [37]. Furthermore, the exclusion of cases with SpA with a previous registered diagnosis of RA precludes a significant misclassification. Regarding COPD, underdiagnosis and misclassification of the disease is still a problem in Sweden [34], and may have resulted in lower incidence estimates. However, this problem is unlikely to be differential between SpA cases and controls and thus rather unlikely to have inflated our incidence ratio estimates. Moreover, the purpose of the study was to estimate the incidence of clinically diagnosed COPD in SpA and not the actual incidence of the disease, including cases that had not come to clinical attention.

Another limitation is that data on smoking and other COPD-related exposures and their distribution between SpA cases and controls are not available in our study. In some settings the prevalence of current smoking in patients with SpA is reported to be higher compared with the general population [38], but this cannot be extrapolated to different settings and to SpA cohorts with different sociodemographic characteristics. Although the relation between smoking and SpA is complex, there is some evidence that smoking is associated with more severe disease [39]. Our previous study of an inception cohort of patients with early axSpA indicated that smokers may have higher disease activity over time and there was an association for smoking with worse spinal pain and morning stiffness over time, adjusted for age, sex, level of education, other lifestyle factors and time-varying treatment for axSpA [40]. Consequently, the benefits of lifestyle modification, including smoking cessation, in patients with SpA are widely recognized and likely reflected in advice given by physicians, healthcare professionals and patient support groups. We cannot thus exclude that differences in the prevalence of risk factors for COPD between cohorts and lifestyle changes following the SpA diagnosis may have inflated the COPD incidence estimates in the present study.

The register-based character of the study did not allow for availability of important information regarding our SpA cohort, such as the HLA-B27 status, the classification as predominantly axial or peripheral disease, or as radiographic/non-radiographic disease in case of axial SpA. Consequently, we could not perform any analysis based on specific SpA subgroups. In addition, data on body mass index were not available.

Furthermore, outcome data in this study were only available through 2018. Further studies will be useful, although recent changes in the relation between SpA and COPD are unlikely.

Finally, the follow-up in some SpA cases started years after the initial SpA diagnosis which may predispose to survivorship bias as more severe SpA cases may have died before the start of follow-up. However, the proportion of SpA cases that died before the start of follow-up (27/1349 = 2.0%) was lower than in controls (163/6744 = 2.4%).

In conclusion, the incidence of COPD was not higher in patients with SpA compared to matched population-based references in this community-based study, which taken together with our previous study results of a similar prevalence of COPD in patients with SpA and population controls, suggests that there may not be an association between SpA and COPD. However, further studies in other countries and settings are needed.

## Supplementary Information

Below is the link to the electronic supplementary material.

ESM 1 (DOCX 22.3 KB)

## Data Availability

The datasets generated and/or analyzed during the current study are not publicly available due to Swedish law but a limited dataset containing data that support the main analyses are available from the corresponding author on reasonable request.
