# Supplementary material for: The incidence of clinically diagnosed chronic obstructive pulmonary disease in patients with spondyloarthritis compared to matched population-based references: a register-based study
Source: Clin Rheumatol. 2026 Sep 2;45(11):6679–87. doi: 10.1007/s10067-026-08382-7 (PMC13723469; doi:10.1007/s10067-026-08382-7)
Supplement: Supplementary file 1 [file 10067_2026_8382_MOESM1_ESM.docx]

**The incidence of clinically diagnosed chronic obstructive pulmonary disease in patients with spondyloarthritis compared to matched population-based references: a register-based study**

Sofia Exarchou^1^, Ankita Sharma^1^, Petros Zamout^1^, Carl Turesson^1^

^1^ Rheumatology, Department of Clinical Sciences Malmö, Lund University, Malmö, Sweden

**Corresponding author:**

Name: Sofia Exarchou

Address: Department of Clinical Sciences Malmö, Rheumatology, Lund University,

Jan Waldenströms gata 1B, 205 02, Malmö, Sweden.

E-mail: sofiaexar@hotmail.com; Phone (mobile): +46(0)765680331

ORCiD ID: <https://orcid.org/0000-0002-9261-7192>

**Online Resource 1**

Overview of the International Classification of Diseases (ICD)-10 codes used for the identification of chronic obstructive pulmonary disease (COPD) and spondylarthritis (SpA) diagnosis

| **Diagnosis** | **ICD-10 code** |
| --- | --- |
| COPD | J41, J42, J43, J440, J441, J448, J449 |
| SpA | M459, M460, M461, M468, M469 |

**Online Resource 2**

Overview of ICD-10 codes used for identification of extra-musculoskeletal manifestations and comorbidities

| **Diagnosis** | **ICD-10 code** |
| --- | --- |
| Anterior uveitis | H20, H221 |
| Psoriasis | L40 |
| Inflammatory bowel disease | K50, K51 |
| Cardiovascular disease | I05, I06, I07, I08, I20, I21, I22, I23, I24, I25, I27, I28, I34, I35, I36, I37, I50, I60, I61, I62, I63, I64, I65, I66, I67.0, I67.1, I67.2, I67.4, I68, I69, I70, I71, I72, I73.1, I73.9B, I73.9X, I74, I79, K55.0, K55.1, K55.9 |
| Diabetes mellitus | E10, E11, E12, E13, E14, O24 |
| Venous thromboembolism | I26, I80, I81, I82 |
| Arrhythmia | I47, I48, I49 |

**Online Resource 3**

Anatomical Therapeutic Chemical (ATC) codes used for the identification of treatment use from the Swedish Prescribed Drug Register

| **Drug class** |  | **ATC** |
| --- | --- | --- |
| Oral glucocorticoids | Prednisolone | H02AB06 |
| NSAIDs | All non-steroidal anti-inflammatory drugs | M01A |
| csDMARDs | Sulfasalazine  Methotrexate | A07EC01  L04AX03 |
| tsDMARDs | Apremilast  JAK inhibitors | L04AA32  L04AA29, L04AA44, L04AA37 |
| bDMARDs | Adalimumab  Certolizumab pegol  Etanercept  Golimumab  Infliximab  Secukinumab  Ixekizumab  Ustekinumab  Guselkumab  Abatacept | L04AB04  L04AB05  L04AB01  L04AB06  L04AB02  L04AC10  L04AC13  L04AC05,  L04AC16  L04AA24 |

NSAIDs: non-steroidal anti-inflammatory drugs; csDMARDs: conventional synthetic Disease Modifying Anti-Rheumatic Drugs; tsDMARDs: targeted synthetic Disease Modifying Anti-Rheumatic Drugs; bDMARDs: biological Disease Modifying Anti-Rheumatic Drugs

**Online Resource 4**

Anatomical Therapeutic Chemical (ATC) codes used for the identification of treatment of COPD from the Swedish Prescribed Drug Register

R03AC, R03BB, R03AK, R03AL, R03DX

**Online Resource 5**. Prevalence of general comorbidities in patients with SpA and controls. Estimates per 1000, with 95% confidence intervals

|  | **SpA** | **Controls** |
| --- | --- | --- |
| Cardiovascular disease | 67 (53-85) | 63 (56-70) |
| Diabetes mellitus | 26 (17-37) | 40 (34-46) |
| Venous thromboembolism | 21 (14-32) | 19 (15-23) |
| Arrythmia | 32 (22-45) | 29 (24-34) |

**Online Resource 6**. Prevalence of general comorbidities in patients with SpA, by incident COPD. Estimates per 1000, with 95% confidence intervals

|  | **No COPD** | **COPD** |
| --- | --- | --- |
| Cardiovascular disease | 64 (50-82) | 132 (43-307) |
| Diabetes mellitus | 25 (16-37) | 26 (1-147) |
| Venous thromboembolism | 19 (12-30) | 26 (1-147) |
| Arrythmia | 32 (23-46) | 26 (1-147) |

**Online Resource 7**

Incidence of Chronic Obstructive Pulmonary Disease in patients with Spondyloarthritis and controls by sex and age-group

|  | SpA cases with incident COPD | Person-yrs at risk | Controls with incident COPD | Person-yrs at risk | SpA | Controls | COPD Incidence Ratio (95% CI) |
| --- | --- | --- | --- | --- | --- | --- | --- |
|  |  |  |  |  | COPD incidence/1000 (95% CI) | COPD incidence/1000 (95% CI) |  |
| Men by Age-group | | | | | | | |
| 17-49 | 2 | 2741.3 | 7 | 12991.0 | 0.73 (0.09-2.64) | 0.54 (0.22-1.11) | 1.35 (0.14-9.05) |
| 50-69 | 14 | 3332.6 | 85 | 16916.5 | 4.20 (2.30-7.05) | 5.02 (4.01-6.21) | 0.84 (0.44-1.48) |
| 70+ | 12 | 787.1 | 41 | 3863.4 | 15.25 (7.88-26.63) | 10.61 (7.62-14.40) | 1.44 (0.69-2.77) |
| Total | 28 | 6861.0 | 133 | 33771.0 | 4.08 (2.71-5.90) | 3.94 (3.30-4.67) | 1.04 (0.66-1.56) |
| Women by Age-group | | | | | | | |
| 17-49 | 2 | 2214.7 | 14 | 10945.2 | 0.90 (0.11- 3.26) | 1.28 (0.70-2.15) | 0.71 (0.08-3.29) |
| 50-69 | 8 | 2224.0 | 56 | 11483.8 | 3.60 (1.55-7.09) | 4.88 (3.68-6.33) | 0.74 (0.30-1.55) |
| 70+ | 0 | 489.7 | 17 | 2631.4 | ND | 6.46 (3.76-10.34) | ND |
| Total | 10 | 4929.9 | 87 | 25066.4 | 2.03 (0.97-3.73) | 3.47 (2.78-4.28) | 0.58 (0.27-1.12) |
